# Case Report: Electrophysiological characteristics of the pelvic floor in spinal epidural lipomatosis

**DOI:** 10.3389/fresc.2025.1669870

**Published:** 2025-10-20

**Authors:** Jiaqian Li, Bin Chen, Hong Jiang, Weishuyi Ruan, Jianhua Li

**Affiliations:** ^1^ Pelvic Floor Dysfunction Diagnosis, Treatment and Rehabilitation Center, Sir Run Run Shaw Hospital, Zhejiang University School of Medicine, Hangzhou, China; ^2^ Department of Rehabilitation Medicine, Sir Run Run Shaw Hospital, Zhejiang University School of Medicine, Hangzhou, China

**Keywords:** spinal epidural lipomatosis, electrophysiology, sympathetic skin response, somatosensory evoked potential, bulbocavernosus reflex latency, pelvic floor

## Abstract

Electrophysiological examination of the pelvic floor plays a crucial role in localizing nerve damage in pelvic floor dysfunction (PFD). Spinal epidural lipomatosis (SEL) is a space-occupying disease of the spinal canal. SEL can cause spine-related symptoms. We report a case of SEL with pelvic floor dysfunction symptoms and provide two sets of pelvic floor electrophysiological data, before and after disease progression. This case highlights the potential utility of electrophysiological assessment in the early diagnosis and monitoring of SEL.

## 1 Background

Neuroelectrophysiological examinations can provide preliminary localization of nerve damage in pelvic floor dysfunction (PFD). Examination methods include, but are not limited to, the bulbocavernosus reflex (BCR), somatosensory evoked potential (SSEP), and sympathetic skin response (SSR). Herein, we describe a case of a patient with PFD. The patient underwent two pelvic floor electrophysiological evaluations spaced 3 months apart. The first examination only found abnormalities in the SSR of the pudendal nerve and the BCR. The second examination revealed abnormal SSEPs of the dorsal penile nerve and neurogenic damage to the urethral sphincter. Finally, imaging identified increased epidural fat at the L5/S1 level. Spinal epidural lipomatosis (SEL) is an intraspinal space-occupying disease with non-specific clinical symptoms (1). This article reports a case of SEL with pelvic floor dysfunction as the initial symptom and emphasizes the potential value of pelvic floor electrophysiological examinations for early diagnosis and monitoring.

## 2 Case study materials

The patient was a 23-year-old man with a 3-year history of recurrent pain in the saddle area after prolonged urinary retention. Regarding his medical history, 3 years prior to his examination, the patient failed to urinate in a timely manner after drinking water multiple times, and gradually developed urinary weakness from repeatedly holding urine. These symptoms persisted for 1 year and gradually developed into saddle area pain, with persistent soreness. These symptoms were not accompanied by lower limb movement or sensory abnormalities. There was no similar medical history among the patient’s family members. Due to his long-term pain, the patient experienced depressive symptoms, such as low mood and decreased appetite, 2 years before this examination. The patient had been prescribed sertraline and duloxetine before discontinuing the medication due to his difficulty urinating. The patient had visited multiple hospitals and was considered to have prostatitis. Intravesical injection therapy (lidocaine, heparin, sodium bicarbonate, etc.) was administered, but the symptoms showed no significant improvement (persistent pain in the saddle area). The patient underwent electrophysiological examinations in the electromyography room of our hospital in August 2024. The examination protocols were as follows: (1) BCR: Electrical stimulation was performed near the coronal sulcus of the penis. Bilateral responses from the ischiocavernosus muscles behind the scrotum were recorded using concentric needle electrodes. The wave width of the stimulation pulse was 0.2–0.5 ms, and the stimulation intensity was seven times the sensory threshold. The signals were recorded with a bandpass filter of 20 Hz–2 kHz and a sampling period of 100–150 ms. The waveforms were considered valid if they were reproducible at least three times under identical stimulation conditions. (2) External urethral sphincter reflex: Electrical stimulation was performed near the coronal sulcus of the penis, and bilateral urethral sphincter reflexes were recorded using concentric needle electrodes. The stimulation and recording parameters were identical to those used for the BCR. (3) External anal sphincter reflex: Electrical stimulation was performed near the coronal sulcus of the penis, and bilateral external anal sphincter reflexes were recorded using concentric needle electrodes. The stimulation and recording parameters were consistent with the above protocols. (4) SSEP: SSEPs were recorded from the pudendal nerve, dorsal penile nerve, and inferior anal (rectal) nerve. The recording electrodes were placed at Cz (−) and Fz (+) on the top of the head, and stimulated on the left/right pubic symphysis, near the coronal sulcus of the penis, and on the perianal skin. The stimulation current lasted for 0.1 ms, and the sensory stimulation was more than three times the amount. The stimulation was repeated 100 times, with a stimulation frequency of 2.9 Hz. The recorded parameters were 20 Hz–3 kHz, and the sampling period was 100 ms. (5) Pudendal nerve skin sympathetic reflex (P-SSR) and perianal skin sympathetic reflex: The recording electrodes were placed near the coronal sulcus of the penis and around the anus. The stimulation site was located at the horizontal line of the right wrist. A 20 mA, 0.2-ms single-pulse stimulus was applied. The recording parameters included a bandwidth of 0.1–100 Hz and a sampling duration of 10 s. (6) Median nerve skin sympathetic reflex (M-SSR): The stimulation site was located at the horizontal line of the right wrist, and the recording sites were in the center of the patient’s palm and foot. The recording parameters and stimulation duration were the same as for the P-SSR. (7) Spontaneous potential and motor unit potential (MUAP): A total of 10–30 potential waveforms were recorded from the external urethral sphincter and external anal sphincter. Spontaneous activity was recorded with the patient in a relaxed state, while MUAP, the duration, percentage of polyphasic waves, average amplitude, and the recruitment pattern during maximal voluntary contraction were assessed.

**Figure 1:**
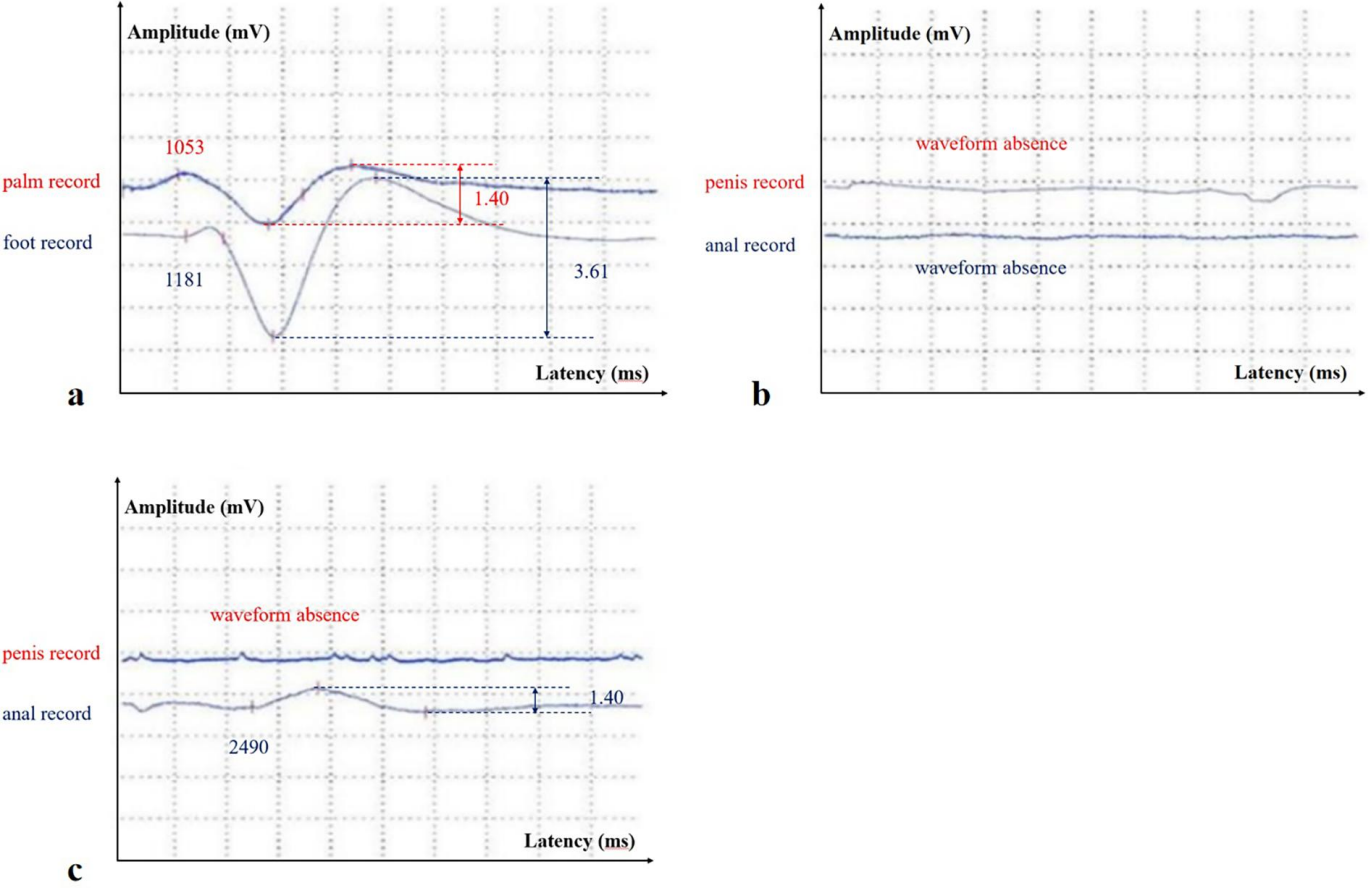
**(a)** The stimulation site was located on the horizontal line of the right wrist, and the recording sites were in the center of the palm and foot, respectively (M-SSR in August 2024); **(b)** the stimulation site was located on the horizontal line of the right wrist, and the recording sites were near the coronal sulcus of the penis (the above waveform) and around the anus (the below waveform) (pudendal nerve SSR and perianal SSR in August 2024); **(c)** the stimulation site was located on the horizontal line of the right wrist, and the recording sites were near the coronal sulcus of the penis (the above waveform) and around the anus (the below waveform) (pudendal nerve SSR and perianal SSR in November 2024).

**Figure 2:**
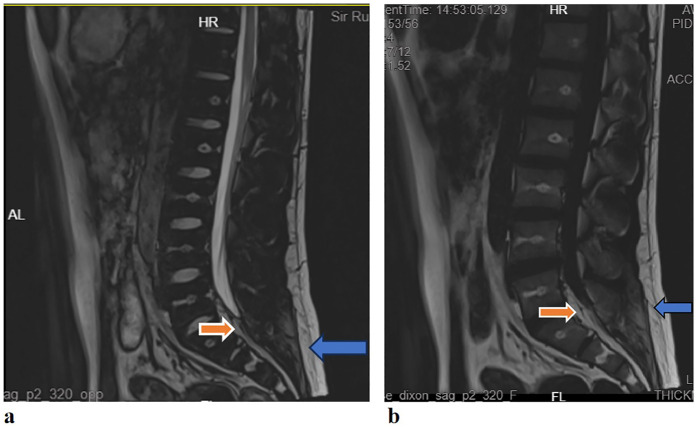
**(a)** Lumbar sacral MRI (T2WI): the sagittal plane showed a high signal behind the L5/S1 cone in the spinal canal (red arrow), which was the same as the fat signal (blue arrow); **(b)** lumbar sacral MRI [T2 fluid attenuated inversion recovery (FLAIR)]: the sagittal plane showed high signal behind the L5/S1 cone in the spinal canal (red arrow), which was the same as the fat signal (blue arrow).

The results of the neuroelectrophysiological examinations revealed an abnormal SSR waveform in the pudendal nerve and prolonged BCR latency (Table 1 and Figure 1). The pudendal nerve SSR indicated extremely low sympathetic excitability. In addition, the patient reported a long-standing habit of holding urine. Combined with the electromyographic results, a partial injury to the pelvic nerve plexus was suspected, and treatment with mecobalamin was initiated. Subsequently, the patient developed tightness in the right pelvic floor muscles, and the symptoms gradually developed into decreased sensation in the sellar region, with frequent urination and urinary leakage. The patient returned to our hospital for inpatient treatment 3 months later (November 2024). A physical examination showed normal muscle strength and tension in all limbs; a decrease in acupuncture sensation around the anus and right saddle area; positive tenderness in the right gluteus medius, the upper middle segment of the iliotibial tract, and the piriformis muscle; and a decrease in deep pressure sensation on the right side during the anal examination. The right hamstring, rectus femoris, and quadratus lumborum muscles were tense, as were the left iliopsoas and rectus femoris muscles. There was no tension or tenderness in his other muscles. Furthermore, the patient’s bilateral knee reflexes and tendon reflexes were normal. He was negative for bilateral ankle spasms, and his bilateral Babinski signs were also negative. The patient’s visual analog scale (VAS) score was 2. An ultrasound of the urinary system indicated fullness of the prostate gland. The residual urine volume examination of the bladder indicated a residual urine volume of approximately 2 mL after urination. The patient underwent pelvic floor electrophysiological examinations again, and the results showed that the injury had progressed. In addition to the previously observed abnormal pudendal nerve SSR waveform (Figure 1) and prolonged BCR latency, there was also a prolonged SSEP latency (prolonged SSEP latency of the dorsal penile nerve) and a slightly elevated systolic potential in the patient’s right urethral sphincter (Table 1). Taken together, these electrophysiological findings suggested a broad neural injury. Combined with the patient’s abnormal BCR and SSEP, there was suspicion of a high possibility of sacral nerve injury and partial injury to the pelvic nerve plexus. Lumbar and sacral MRIs were recommended. The enhanced lumbar plexus MRI examination showed increased epidural fat at the L5/S1 level, occupying approximately 50% of the spinal canal (Figure 2). Based on his medical history, physical examinations, and imaging results, the patient was ultimately diagnosed with SEL. During hospitalization, the patient mainly received rehabilitation treatment (extracorporeal shock wave therapy, electroacupuncture, biofeedback, and transcranial magnetic stimulation). After 10 days of treatment, the patient refused to continue the treatment due to significant emotional fluctuations and was discharged.

**Table 1:** Pelvic floor electromyography results.

Results			Normal value		August 2024		November 2024	
			Latency (ms)	Amplitude (mV)	Latency (ms)	Amplitude (mV)	Latency (ms)	Amplitude (mV)
SSEP	Dorsal penile nerve SSEP		35.1 ± 1.7	–	31.3	–	44.3	–
	Pudendal nerve SSEP		35.4 ± 2.1	–	35.3	–	39.8	–
	Inferior anal nerve SSEP		35.5 ± 0.8	–	37.8	–	38.7	–
Reflex	External urethral sphincter reflex (left/right)		27.9 ± 2.1	–	25.3/25.5	–	24.5/23.6	–
	BCR (left/right)		27.5 ± 1.7	–	34.2/32.4	–	34.7/36.5	–
	External anal sphincter reflex (left/right)		27.0 ± 1.8	–	23.9/24.4	–	25.1/27.2	–
SSR	M-SSR (palm record)		1,410 ± 130	2.19 ± 1.14	1,053	1.40	–	–
	M-SSR (foot record)		2,020 ± 160	0.55 ± 0.28	1,181	3.61	–	–
	P-SSR (penis record)		1,438 ± 49	–	Absence	Absence	Absence	Absence
	P-SSR (anal record)		1,444 ± 79	–	Absence	Absence	2,490	0.30
Spontaneous potential and MUAP	External urethral sphincter	Spontaneous potential	No		No		No	
		MUAP	Normal (bilateral)		Normal (bilateral)		Normal (left)/Large (right)	
	Bulbocavernosus muscle	Spontaneous potential	No		No		–	
		MUAP	Normal (bilateral)		Normal (bilateral)		–	
	External urethral sphincter	Spontaneous potential	No		No		No	
		MUAP	Normal (bilateral)		Normal (bilateral)		Normal (bilateral)	

**Figure 3:**
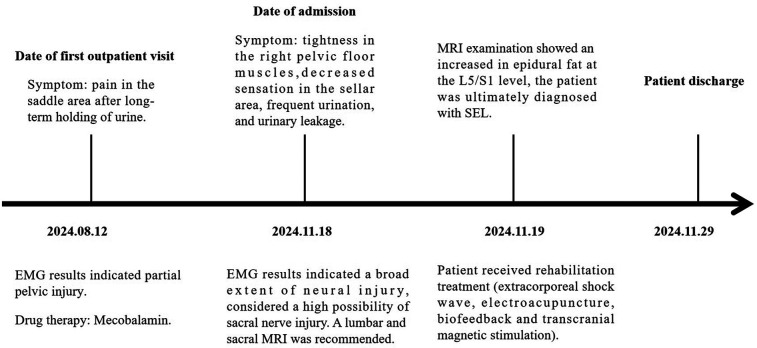
The main recorded changes during the first outpatient visit and the patient's hospital stay from the first day of admission.

The patient’s timeline is presented in Figure 3 and illustrates the examinations conducted from the first outpatient visit to admission, hospitalization, and discharge.

## 3 Discussion

**Figure 4:**
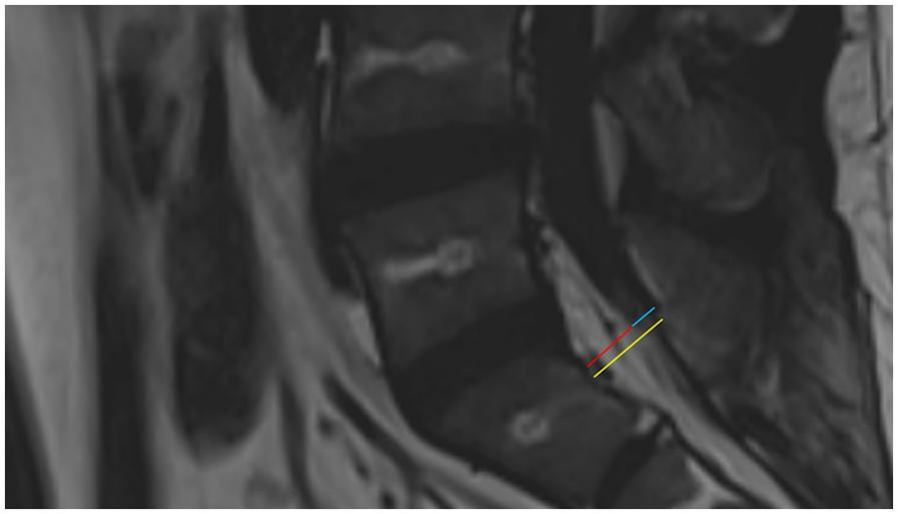
Sagittal T2 FLAIR MRI of the patient. The yellow line shows the anteroposterior maximum diameter of the spinal canal, the red line shows the anteroposterior maximum diameter of the epidural fat, and the blue line shows the anteroposterior maximum diameter of the dural sac.

SEL is a neurological condition caused by excessive adipose tissue within the spinal canal compressing the surrounding dura mater and nerve structures. Its clinical symptoms lack specificity and may include chronic pain in the lower back and limbs, numbness in the lower limbs, and decreased sensation. In severe cases, SEL can lead to cauda equina syndrome or even paralysis (2). Common causes currently include long-term use of exogenous steroids, exposure to endogenous steroids, obesity, surgical induction, and idiopathic diseases (3, 4). Diagnosis primarily relies on MRI, which is considered the gold standard(5). The thickness of epidural fat in normal individuals is usually between 3 and 6 mm in the sagittal plane (6). Objective grading of excess epidural fat in patients with lumbar SEL is usually based on the MRI grading proposed by Borré et al.in 2003 (7). Grading is performed on an axial plane that is parallel and tangent to the superior endplate of the vertebral body, and the following three measurement values are obtained: anteroposterior maximum diameter of the dural sac (DuS), anteroposterior maximum diameter of the epidural fat (EF), and anteroposterior maximum diameter of the spinal canal (SC). With these values, the DuS/EF and EF/SC indices can be calculated. Details of the grading are shown in Table 2 (7, 8). In this case, the patient’s measurements for SC, EF, and DuS were 15.48 mm, EF: 10.40 mm, DuS:5.08 mm, respectively, resulting in calculated DuS/EF and EF/SC indices of 0.49 and 67%, respectively. Thus, according to the classification criteria, the patient’s SEL was Grade II (Figure 4). This report highlights the pelvic floor electrophysiological findings in SEL and documents two sets of electrophysiological data corresponding to different stages of disease progression.

**Table 2:** MRI grading of lumbar SEL proposed by Borré.

MRI grade	DuS/EF index	EF/SC index (%)	Meaning
Grade 0 (normal)	≥ 1.5	<40%	Normal amount of epidural fat
Grade I	1.49–1	41%–50%	Mild overgrowth of epidural fat
Grade II	0.99–0.34	51%–74%	Moderate overgrowth of epidural fat
Grade III	0.33	≥75%	Severe overgrowth of epidural fat

DuS, anteroposterior maximum diameter of the dural sac; EF, anteroposterior maximum diameter of the epidural fat; SC, anteroposterior maximum diameter of the spinal canal.

Electrophysiological examinations of the pelvic floor can help localize the site of a nerve injury. In this case, symptoms of pelvic floor nerve dysfunction developed progressively within 3 months. During the first electromyography examination, the patient's clinical symptoms mainly manifested as pain in the saddle area. Pelvic floor electromyography only revealed low excitability of the pudendal nerve SSR and abnormal BCR. The SSR has been used to evaluate the function of postganglionic sympathetic fibers, and changes in this response have been related to the activity of the sweat glands (9). Since the SSR is a form of reflex arc, damage to any part of the conduction pathway may result in an abnormal SSR. We simultaneously performed median nerve electrical stimulation on the patient, and the SSR waveform recorded in the palm/foot showed a shortened latency (Figure 1), indicating high excitability. This further suggested that the damage to the conduction pathway originated locally rather than systemically. BCR refers to the contraction of the bulbocavernosus muscle in response to stimulation of the pudendal nerve. It is mediated through a sacral reflex arc, and its latency reflects the integrity of the sensory afferent nerves (pudendal nerves), the sacral spinal cord (S2–S4), and efferent motor fibers (10). The patient's symptoms had progressed 3 months later to decreased sensation in the sellar region, frequent urination, and urinary leakage. Further pelvic floor electrophysiological examinations showed an extended SSEP latency in the dorsal penile nerve and a large, slightly systolic potential in the urethral sphincter. SSEP is the cortical potential recorded by stimulating the pudendal nerves and propagating through the spinal cord, and reflects the integrity of the somatosensory pathway. Previous studies have shown that central nervous system diseases are mainly characterized by SSEP abnormalities alone, while peripheral nervous system diseases are characterized by both BCR and SSEP abnormalities. The patient had abnormalities in BCR and SSEP, located in the nerves of the sacral segment and below. Needle electromyography suggested involvement of the motor branch of the pudendal nerve, which belongs to the sacral plexus nerve branch. Ultimately, we considered that there was a high possibility of sacral nerve injury in the patient. We confirmed peripheral nerve compression caused by increased L5/S1 epidural fat through lumbar plexus enhanced magnetic resonance imaging. In this case, an abnormal SSR of the autonomic nervous system occurred earlier than other electromyographic changes or even pelvic floor dysfunction symptoms in the patient. This phenomenon has also been reported in familial amyloidosis polyneuropathy (FAP) (11). FAP is a familial disease, and the first symptom in male patients may be sexual dysfunction. In a previous study of 15 patients with FAP, the pudendal nerve SSR parameter was the most sensitive indicator throughout disease progression. This may be related to the greater contribution of the autonomic nervous system to the innervation of the genitourinary tract compared to the somatic nerves (12). This case also suggested that the pudendal nerve SSR may be more sensitive than other electrophysiological measures in detecting early pudendal nerve injury. We therefore recommend performing pudendal nerve SSR examinations in patients with pudendal nerve-related symptoms to improve early diagnostic accuracy.

## Data Availability

The raw data supporting the conclusions of this article will be made available by the authors, without undue reservation.

## References

[1] Zhai S.. One case of idiopathic epidural steatosis with literature review. (n.d.). 10.3969/j.issn.1005-7234.2020.04.047 (Accessed December 8, 2024).

[2] Yasuda T Suzuki K Kawaguchi Y Seki S Makino H Watanabe K Clinical and imaging characteristics in patients undergoing surgery for lumbar epidural lipomatosis. BMC Musculoskelet Disord. (2018) 19:66. 10.1186/s12891-018-1988-8 29490659 PMC5831840

[3] Kim K Mendelis J Cho W. Spinal epidural lipomatosis: a review of pathogenesis, characteristics, clinical presentation, and management. Global Spine J. (2019) 9:658–65. 10.1177/2192568218793617 31448201 PMC6693071

[4] Fogel GR Cunningham PY Esses SI. Spinal epidural lipomatosis: case reports, literature review and meta-analysis. Spine J. (2005) 5:202–11. 10.1016/j.spinee.2004.05.252 15795966

[5] Ferlic PW Mannion AF Jeszenszky D Porchet F Fekete TF Kleinstück F Patient-reported outcome of surgical treatment for lumbar spinal epidural lipomatosis. Spine J. (2016) 16:1333–41. 10.1016/j.spinee.2016.06.022 27363757

[6] Kuhn MJ Youssef HT Swan TL Swenson LC. Lumbar epidural lipomatosis: the “Y” sign of thecal sac compression. Comput Med Imaging Graph. (1994) 18:367–72. 10.1016/0895-6111(94)90007-8 7954313

[7] Borré DG Borré GE Aude F Palmieri GN. Lumbosacral epidural lipomatosis: MRI grading. Eur Radiol. (2003) 13:1709–21. 10.1007/s00330-002-1716-4 12835988

[8] Mh T Hm C Km M Cy C. Unrecognized synergistic effect of spinal epidural lipomatosis on spinal stenosis and its radiological grading: a case series. Cureus. (2025) 17:e87439. 10.7759/cureus.87439 40772216 PMC12327442

[9] Hu W Cheng Y Pan J Wang X Li S Fan Z Value of electrophysiological indicators in differential diagnosis of Parkinson’s disease and multiple system atrophy. BMC Neurol. (2023) 23:94. 10.1186/s12883-023-03131-8 36864385 PMC9979443

[10] Li X Wang C Zhang X Zhang W Deng B Wang X The value of sacral reflex and sympathetic skin reflex in the diagnosis of multiple system atrophy P-type. Parkinsons Dis. (2021) 2021:6646259. 10.1155/2021/6646259 33552462 PMC7843193

[11] Alves M Conceição I Luis ML. Neurophysiological evaluation of sexual dysfunction in familial amyloidotic polyneuropathy–Portuguese type. Acta Neurol Scand. (1997) 96:163–6. 10.1111/j.1600-0404.1997.tb00260.x 9300069

[12] Opsomer RJ Guérit JM Van Cangh PJ Zarola F Romani GL Rossini PM. Electrophysiological assessment of somatic nerves controlling the genital and urinary functions. Electroencephalogr Clin Neurophysiol Suppl. (1990) 41:298–305. 10.1016/b978-0-444-81352-7.50035-2 2289442

